# Construction and Validation of the 21 Item Fitness-to-Drive Screening Measure Short-Form

**DOI:** 10.3389/fpubh.2018.00339

**Published:** 2018-12-06

**Authors:** Sherrilene Classen, Shabnam Medhizadah, Sergio Romero, Mi Jung Lee

**Affiliations:** ^1^Department of Occupational Therapy, University of Florida, Gainesville, FL, United States; ^2^Center of Innovation on Disability & Rehabilitation Research, United States Department of Veterans Affairs, Gainesville, FL, United States

**Keywords:** aging, proxy raters, decision support system, automobile driving, fitness to drive

## Abstract

**Introduction:** The Fitness-to-Drive Screening Measure is a free online screening tool that detects at-risk older drivers, however, it's 20 min administration time may render the 54-item tool less than optimal for clinical use. Thus, this study constructed and validated a 21-item FTDS Short-Form (FTDS-SF).

**Method:** This mixed methods study used 200 proxy rater responses and older driver on-road assessments. We conducted a Rasch analysis to examine information at the level of the item and used content validity index scores to select items. Using a receiver operator characteristics curve we determined the concurrent validity of the FTDS-SF to on-road outcomes.

**Results:** Twenty-one items were selected for the FTDS-SF. The area under the curve = 0.72, indicated the FTDS-SF predicted on-road outcomes with acceptable accuracy. Still, 68 drivers were misclassified.

**Conclusion:** The FTDS-SF may reduce administration time, while still yielding acceptable psychometric properties. Yet, caution needs to be executed in clinical decision making as the measure is overly specific.

## Introduction

Clinicians may benefit from an efficient and valid screening measure to detect at-risk older drivers and to help prevent the risk of crash-related deaths or injuries. The existing web-based Fitness-to-Drive Screening Measure (FTDS) has the potential to serve such a purpose, but its length currently limits its uptake. The purpose of this study is to construct and validate an FTDS Short-Form (FTDS-SF) that may aid clinicians in screening older drivers, detecting their crash-risk and identifying mitigation strategies to help reduce motor vehicle fatalities or serious injuries while also protecting the public's health in North America.

## Background

By 2020, ~40 million (18%) license holders in the U.S. and 6 million (23%) in Canada will be classified as older drivers over the age of 65 (1, 2). Strikingly, despite older drivers being the likeliest group to observe safe driving practices, including using seat belts, driving under safe conditions, and avoiding driving under the influence of alcohol, they are the second most prevalent group involved in motor vehicle crashes (3–6). Although older drivers adhere to safe driving practices, their increased risk for injury or death in motor vehicle crashes stem from age-related declines in visual, cognitive, motor, and other sensory functions that impact their ability to drive safely (7). Age-related factors impacting older adults' fitness to drive include the ability to control a vehicle while conforming to the rules of the road and obeying traffic laws, declines in vision and reaction times, and decreased function in reasoning and recall (7–9). As the number of older adults over 65 years increases in North America, screening drivers becomes a critical factor in avoiding crashes by detecting and classifying drivers' crash risks and identifying mitigation strategies.

Current drivers' licensing laws typically require only visual tests or quizzes concerning traffic safety laws. In some U.S. states, older drivers may exercise an option to renew licenses issued for 8 years by mail or online, resulting in 16 years where no official assesses an older driver's fitness to drive (10). Typically, clinicians (e.g., primary care physicians, specialists such as geriatricians or neurologists, nurse practitioners, physician's assistants, and occupational therapists) become the only gatekeepers who systematically screen older adults' driving fitness (11). However, many clinicians report inadequate training and/or confidence in their ability to evaluate driver fitness (12–16) and as such, miss an important opportunity to detect at-risk older drivers and to implement risk mitigation strategies. The FTDS aids clinicians with initial risk detection and classification strategies by providing a results summary that has calibrated client data collected through proxy report. The FTDS also provides recommendations and resources to mitigate driver risk (17).

The FTDS, a free, web-based screening tool with established criteria validity and reliability, identifies at-risk older drivers by using a proxy rater that includes family members, close friends or caregivers who have been passengers in the past 3 months—and thus able to provide objective responses to the questions (17–23). The FTDS screening takes approximately 20 min to complete and contains three sections: demographic items about the driver and proxy rater; items about the driver's driving history; and 54 items addressing driving behaviors. Proxy raters use a four-point scale to rate the items ranging from *not difficult* (e.g., how difficult a driver finds staying in the proper lane) to *very difficult* (e.g., how challenging a driver finds driving in a rainstorm). The FTDS then generates one of three driver-risk classifications: *At-risk driver:* critical safety concerns exist that must be addressed immediately; *Routine driver:* some safety concerns exist with early signs of needing intervention; or *Accomplished driver:* no safety concerns present. The FTDS also produces a keyform, or driver-risk profile, indicating the probability (logit score with cut-points) that the driver can safely perform the 54 items of the FTDS, all central to avoiding crashes. Based on the specific driver-risk classification, the FTDS provides risk mitigation resources including a listing of all driver rehabilitation specialists and recommendations such as initiating conversations about driving cessation. The FTDS completed by proxy raters are used in driving assessments by occupational therapists but is lengthy and time-consuming to complete (24).

In prior work, the FTDS (formerly called the Safe Driving Behavior Measure or SDBM) has undergone psychometric testing to indicate that it is a valid and reliable screening method for identifying at-risk older drivers (17). Using Google analytics (n.d.), the patterns and trends of FTDS users was explored and established (25). Specifically, we determined that although over 43,000 users have accessed the FTDS, they failed to spend the recommended 20 min to complete the FTDS. To overcome this issue, our research team recommended decreasing the completion time of the FTDS by reducing the number of items, thus, the FTDS underwent item reduction (26). Using classical test theory rather than item response theory, the 54-item FTDS was reduced to a 32-item version. Validity testing of the 32-item FTDS indicated excellent concurrent validity with the 54-item FTDS (*r* = 0.99) (26). ROC curve results indicated the 32-item version could correctly discriminate between drivers who passed or failed the on-road assessment (AUC = 0.75, *p* < 0.05, 95% CI [0.65, 0.84]; Medhizadah, Classen, & Johnson, submitted). This method provides results in a metric different than the Rasch based FTDS. To ensure consistency across FTDS forms and to take advantage of the added benefits of Rasch, such as developing keyforms, disease –specific forms and computer adaptive versions, we opted to construct a Rasch based FTDS-SF.

### Rationale, Significance, and Purpose

While the FTDS can detect at-risk older drivers, it is lengthy in administration (25). If we develop and implement an FTDS-SF, it will gain the FTDS wider uptake among proxies and clinicians. The Rasch framework provides the statistical rigor to accurately reduce redundant items while optimizing the measurement precision necessary for an FTDS-SF (27, 28). Therefore, we used the Rasch methodology because of its robustness, simplicity, and parsimony to create an FTDS-SF from the Rasch-calibrated 54-item FTDS. We solicited clinician input to verify the clinical appropriateness of the items, and we determined the concurrent predictive validity of the FTDS-SF to on-road outcomes.

## Methods

The University of Florida's Institutional Review Board approved this secondary data analysis as a board review exemption on July 22nd, 2016. The de-identified data used in this study was previously collected for the primary FTDS study (17). The primary study was approved by the University of Florida's Institutional Review Board and all the participants, proxies (*n* = 200) and drivers (*n* = 200) provided informed consent. Drivers received USD 100 and proxy raters received USD 50 for their participation.

### Design

This secondary data analysis used the embedded concurrent and sequential mixed method design. Rasch analysis was used to determine the critical items and the qualitative content validity method was used to verify the clinical relevance of these items. We used a receiver operator characteristics (ROC) curve to determine the concurrent validity of the FTDS-SF to on-road outcomes.

### Participants

In the primary study 200 older drivers and 200 of their proxy raters were recruited from North Central Florida and Thunder Bay, Ontario, Canada via newspaper advertisements, word-of-mouth referrals and flyer distribution.

#### Older Drivers

The community-dwelling older drivers were between the ages of 65–85, driving at the time of recruitment with a valid driver's license, and had the physical ability to complete an on-road assessment. Older drivers were excluded if they were medically advised not to drive, experienced uncontrolled seizures, or used medications that impaired their central nervous system.

#### Proxy Raters

Proxy raters (family members, friends, formal/informal caregivers) were included if they were between the ages of 18–85, had observed the older driver's driving behaviors in the last three months and were able to report on those behaviors. Proxy raters were excluded if during screening procedures they exhibited physical or mental conditions that could impair their ability to make observations of the driver's driving behaviors.

#### Content Experts

A convenience sample of three content experts included one driver rehabilitation specialist and two certified driver rehabilitation specialists. Expert 1 was a licensed occupational therapist and certified driver rehabilitation specialist practicing in California with 21 years of experience in driver rehabilitation. Expert 2, a clinical assistant professor from the University of Florida, was also a licensed occupational therapist and certified driver rehabilitation specialist with 8 years of experience with older drivers. Expert 3 was a licensed occupational therapist and driver rehabilitation specialist practicing in Michigan with 2 years of experience with older drivers.

### Procedure

This study followed a three-phase process to develop and validate the FTDS-SF. First, we used Rasch analysis to create an initial pool of items. Next, our content experts rated the items for relevance of determining fitness to drive. The final items were selected and reviewed by the team. Lastly, we examined the concurrent validity of the FTDS-SF to the on-road outcome data of the older drivers.

### Data Management

The de-identified data of the older drivers and proxies, as well as those of the content experts, were stored on a password-protected server network at the University of Florida that adhered to the policies of security, privacy and confidentiality.

#### Proxy Rater Responses

To avoid using items that provided little information, i.e., limited variation in proxy rater responses to FTDS items, a frequency analysis was performed. An FTDS item that had 90% or more of the responses in one of the four rating categories was removed.

#### Content Expert Ratings

Each expert was invited by email to rate the relevance of the Rasch-derived FTDS items. The experts had to critically appraise whether each item from the reduced pool of items identified in the Rasch analysis was essential for determining the fitness to drive of an older adult. They were asked to score each item on a 3-point scale indicating whether the item was; 1 = *not essential*, 2 = *useful but not essential*, 3 = *essential* for determining fitness to drive. After scoring, the content experts returned the ratings to the research team. Lastly, the research team calculated the content validity index of each item (29). Items with no agreement underwent a second round of reviews to finalize the item choices. Next, we dichotomized the rating scale into *relevant* and *not relevant* categories. Thus, the 3-point scale was divided into the r*elevant* category by combining rating 2, i.e., *useful but not essential* and rating 3, i.e., *essential*. The *not relevant* category included the rating of 1, i.e., *not essential*. Using the dichotomized ratings, *relevant* ratings were assigned a value of 1 and *not relevant* ratings were assigned a value of 0.

### Data Analysis

**Figure 1:**
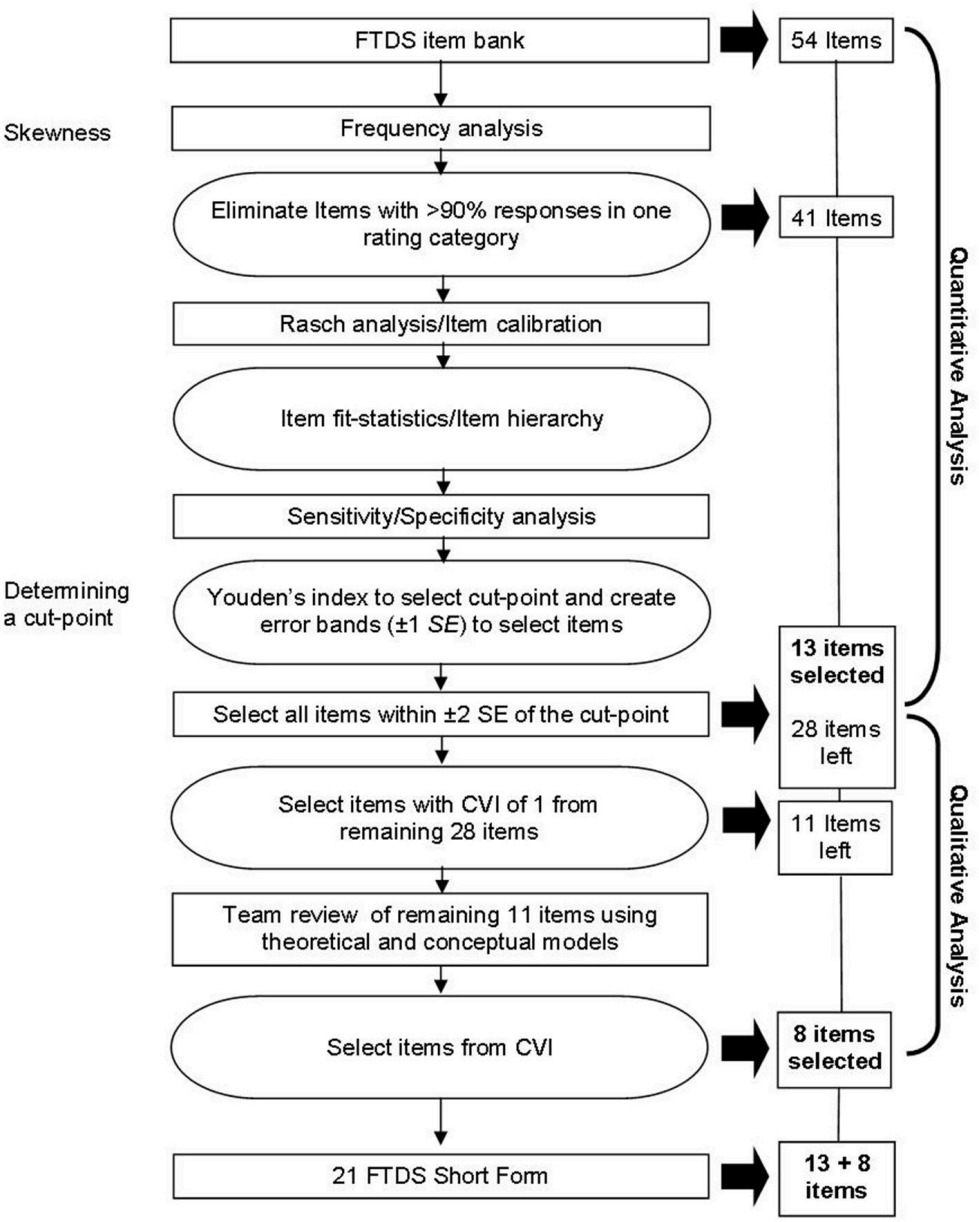
Flowchart of the procedures for constructing the Fitness-to-Drive Screening Measure Short-Form. FTDS, Fitness-to-Drive Screening measure; SE, Standard error; CVI, Content validity index.

First, for the quantitative analysis, we used the procedures outlined by del Toro et al. (30) for developing a short-form of the Boston Naming test. This process includes conducting a frequency analysis (described above), examining unidimensionality, and performing a psychometric analysis using Rasch. Secondly, for the qualitative analysis, we used the content validity index to review candidate items and confirmed their relevance using the extant literature (20) for empirical support (29). A flowchart is presented in Figure 1 to describe the process. Finally, we used a ROC curve, with area under the curve (AUC), sensitivity, specificity, positive predictive value (PPV), negative predictive value (NPV), and misclassifications to establish the concurrent validity of the FTDS-SF.

#### Quantitative Phase

Based on the frequency analysis, we removed items with 90% of rater responses in one category. Using WINSTEP software (4.0.1, 2017) we conducted a Rasch Partial Credit Model analysis on the remaining items (*n* = 41) We examined the infit (information-weighted) and outfit (outlier-sensitive) statistics for model fit. Specifically, infit statistics indicate unexpected patterns of responses for person ability; whereas outfit statistics indicate unexpected rater responses. For a socio-behavioral tool, such as the FTDS, values for infit and outfit mean squares between 0.5 and 2.0 are acceptable (31, 32).

Using Rasch analysis, the items were calibrated on an item difficulty continuum, ordering items from least difficult to most difficult across the driver's ability levels. Item reliability, which is used to verify the item hierarchy and ability to discriminate between items of various difficulty levels, was examined. Person reliability was used to examine the FTDS' ability to reliably distinguish the different levels of ability in our sample.

To select items from the FTDS that were most relevant for screening at-risk drivers we calculated a ROC curve using Rasch person ability estimations and on-road pass/fail outcomes. Through this process we determined the cut-point with maximum discrimination for differentiating between drivers that would pass or fail an on-road assessment. ROC curves graphically demonstrate the ability of the measure to differentiate between two groups (e.g., pass and fails) at all possible cut-points by plotting the sensitivity against 1-specificity. To select the cut-point with maximum discrimination and the least number of misclassifications, we used Youden's index (*J)* (33). Youden's index ranges from 0 to 1, with values closest to 1 indicating high sensitivity and specificity (34). To ensure item coverage of all driving ability levels around the selected cut-point, while also reducing item redundancy, we created error bands at 1 standard error (*SE*) above and below the cut-point. To ensure maximum information for separating older drivers into at least two groups, we included all items in the FTDS-SF within ± 2 *SE* of the cut-point. To select items from the remaining error bands (± 3–5 *SE*), contents experts were asked to rate the relevancy of each item, next described.

#### Qualitative Phase

For the remaining items the item content validity index for relevancy was calculated. The item content validity index is the proportion of agreement on the relevancy of each item, with ranges from from 0 to 1. Specifically, item content validity index is the number of items judged relevant (*useful but not essential* or *essential)* divided by the numbers of content experts (29). We used a content validity index criterion of 1 to select items from the remaining error bands. If no item in an error band met the criterion of 1, the content experts collaboratively decided on its inclusion/exclusion. The scale content validity index, or the proportion of items in an instrument that achieves a rating of *relevant* by the content experts, was calculated (29). A scale content validity index of 0.80 or higher for all items in a measure is considered acceptable (29).

#### Concurrent Validity

We analyzed 200 FTDS-SF proxy rater logit scores to 200 older driver on-road pass/fail outcomes using a ROC curve. First, the AUC which represents the screening measure's ability to differentiate between older drivers who passed/failed the on-road assessment, was examined (33). Next, using *J* index, we determined the cut-point, and its associated sensitivity, specificity, PPV, NPV and total number of misclassifications. Lastly, to determine the logit score thresholds for separating at-risk drivers from routine drivers and routine drivers from accomplished drivers, we used 2 *SE* above and below the cut-point for the FTDS-SF.

## Results

### Participants Demographics

From the 200 older drivers (*M*_*age*_ = 73.64, *SD* = 5.35) and 200 proxy raters (*M*_*age*_ = 62.44, *SD* = 14.76), 165 were male (110 drivers; 55 proxy raters) and 135 were female (90 drivers; 145 proxy raters). One hundred sixty-nine of the 200 drivers passed (84.5%) the on-road assessment. The detailed descriptive profiles of the drivers and proxy raters are published elsewhere (17).

### Quantitative Analysis

Frequency analysis indicated 13 of the 54 items had 90% or more of the rater responses in one category. For all 13 items (i.e., 1, 3, 4, 6, 9, 10, 12, 14, 18, 20, 27, 30, and 36) 90% of the raters responded *not difficult*. Table 1 contains the 13 excluded items by number.

**Table 1:** List of items excluded from the FTDS Short-Form with response rates >90%.

**Item**	**Item description “how difficult is it for him or her to…”**	**Rater responses (%)**
1.	Drive in the proper lane	90.50
3.	Use the vehicle controls	93.00
4.	Check your mirrors when changing lanes	90.50
6.	Obey varied forms of traffic signals	95.00
9.	Drive in light rain	93.00
10.	Drive on a highway with two or more lanes in each direction	93.00
12.	Keep distance from other vehicles when changing lanes?	91.00
14.	Drive cautiously (to avoid collisions) in situations when others are driving erratically?	90.50
18.	Enter the flow of traffic when turning right?	93.00
20.	Drive on graded (unpaved) road?	94.70
27.	Stay within the lane makings unless changing lanes?	92.00
30.	Look left and right before entering an intersection?	96.00
36.	Control his or her car when going down a steep hill	90.40

For all items, 90% or more of the raters responded, “not difficult.”

From the Rasch analysis, the remaining 41 items demonstrated acceptable infit (i.e., 0.5–2.0) and most displayed acceptable outfit (0.5–2.0) mean squares. However, item 2 i.e., *check for a clear path when backing out from a driveway or parking space* (Infit: 1.18, Outfit: 2.49) and item 43, i.e., *stay focused on driving when there are distractions* (Infit: 1.39 and Outfit: 2.17) displayed outfit values >2.0. Because outfit statistics can be highly influenced by the presence of a few outliers, such as unexpected observations that are not representative of the data (31), we did not remove the two items from the item pool. Rasch calibration results for the 41 remaining items indicated a range of item difficulty from 2.47 to 4.24 logits. The easiest item with a logit score of 2.47 was item #19, i.e., *share the road with vulnerable road users*, and the most difficult item with a logit score of 4.24 was item #54, i.e., c*ontrol your car on an icy road*. The item reliability coefficient was 96%, while the person reliability coefficient was 81%.

Youden's index indicated the cut-point with maximum discrimination and the least number of misclassifications was at 3.40 logits, yielding a sensitivity of 0.84 and specificity of 0.55. The 3.40 logit cut-point indicated the center of the error band distribution, with five error bands identified above and below (labeled 1 through 10) this cut-point as indicated in Table 2. Items in error band 1 represented the easiest items and items in error band 10 represented the most difficult items. The two error bands were removed because the goal was to provide the maximum information for discriminating between drivers that would pass or fail an on-road assessment near the cut-point. Items within these two error bands provided the least discriminatory information for separating subjects into two groups (pass or fail). All 13 items from within error bands 4, 5, 6, and 7 (see Table 2) were selected for the FTDS-SF because they were within ± 2 *SE* of the cut-point and provided the most information for separating groups.

**Table 2:** Rasch calibrated FTDS item's error bands, item difficulty, infti/outfit statitistics, item content validity index and items selected for the FTDS Short-Form.

**Item**	**Item description “how difficult is it for him or her to…”**	**Item difficulty (logits)**	**Infit mean square**	**Outfit mean square**	**Error band**	**Item-content validity index**
54.	Drive on an icy road?	4.24	1.10	1.16	10	–
51.	**Drive in a thunderstorm with heavy rains and wind?**	**4.11**	**0.94**	**0.96**		**1.00**
24.	Use a paper map while driving?	4.02	1.81	1.70	9	0.33
49.	**Drive when there is glare or the sun is in his or her eyes?**	**4.01**	**0.97**	**1.37**		**1.00**
48.	**Drive at night on a dark road with faded or absent lane lines?**	**3.92**	**0.98**	**0.92**		**1.00**
38.	**Drive in a highly complex situation?**	**3.90**	**0.70**	**0.74**	8	**1.00**
53.	Drive on a snow covered road?	3.80	1.02	0.94		0.67
35.	Drive in an unfamiliar urban area?	3.60	0.72	0.66	**7**	Selected
44.	Drive in an unfamiliar area?	3.53	0.73	0.73		Selected
47.	Drive when there is fog	3.53	0.80	0.88		Selected
45.	Drive at night	3.48	1.14	1.04	6	Selected
42.	Drive when upset?	3.44	0.85	0.89		Selected
**CUT-POINT (3.40 LOGITS)**						
26.	Parallel park?	3.35	1.43	1.39		Selected
34.	Pass (overtake) a larger vehicle such as an RV, tractor-trailer (transport truck), or dump truck in the absence of a passing lane?	3.29	1.13	0.99	5	Selected
41.	Alter his or her driving in response to changes in health or condition?	3.24	0.97	1.02		Selected
46.	Avoid dangerous situations (such as car door opening)?	3.21	0.78	0.93		Selected
43.	Stay focused on driving when there are distractions?	3.18	0.77	0.72		Selected
50.	Turn left across multiple lanes when there is no traffic signal?	3.14	0.87	0.70	4	Selected
40.	Drive a different car (such as another person's car or a rental car)?	3.13	0.78	0.71		Selected
25.	Make a left-hand turn crossing multiple lanes and entering traffic?	3.06	1.06	0.74		Selected
7.	Drive and hold a conversation with one or more passengers?	3.00	1.23	1.03		0.67
8.	Drive with a passenger who is providing driving directions or assistance?	2.99	1.39	1.67		0.67
16.	Maintain lane when turning (not cut corner or go wide)?	2.99	1.21	1.17		0.67
29.	**Keep distance between your car and others?**	**2.96**	**1.39**	**2.17**		**1.00**
33.	Pass (overtake) another car on a road without a passing lane?	2.94	1.01	0.75	3	0.67
5.	Read road signs far enough in advance to react?	2.92	1.07	1.05		0.67
32.	**Drive in dense traffic (such as rush hour)?**	**2.90**	**0.68**	**0.52**		**1.00**
21.	**Check blind spots before changing lanes?**	**2.89**	**0.99**	**1.11**		**1.00**
37.	**Exit an expressway, or inter-state from a left-hand lane?**	**2.89**	**1.31**	**0.86**		**1.00**
22.	Drive with tractor-trailers (transport trucks)?	2.88	0.94	0.63		0.67
11.	Keep up with the flow of traffic	2.84	1.01	1.03		0.67
39.	Control the car (brake hard or swerve) to avoid collisions?	2.81	0.89	0.55		0.67
52.	**Control his or her car on a wet road?**	**2.80**	**0.70**	**0.53**		**1.00**
17.	Back out of parking spots?	2.78	1.25	1.19		0.67
2.	Check for a clear path when backing out from a driveway or parking space?	2.74	1.18	2.49	2	0.67
28.	Stay within your lane in the absence of road features such as clearly marked lane lines, reflectors or rumble strips?	2.72	0.99	0.67		0.67
31.	**Drive in a construction zone?**	**2.70**	**1.05**	**0.99**		**1.00**
13.	**Change lanes in moderate traffic?**	**2.67**	**0.74**	**0.74**		**1.00**
15.	29. Brake at a stop sign so car stops completely before the marked line	2.61	1.30	1.18		–
23.	Merge onto a highway	2.50	0.93	0.55	1	–
19.	Share the road with vulnerable road users such as bicyclists, scooter drivers, motorcyclists	2.47	0.92	1.54		–

*Items with a content validity index of 1 are bolded in black. Bolded items highlighted in gray represent the 21 items selected for the final FTDS-SF*.

### Qualitative Analysis

The remaining error bands (2, 3, 8, 9), had atleast one item that had a content validity index of one. As shown in Table 2, initially 11 items (i.e., item #13, 21, 29, 31, 32, 37, 38, 48, 49, 51, 52) with an item content validity index of 1 were selected through the content validity approach. This resulted in an item bank of 24 items, with 13 items selected from the quantitative phase and 11 items from the qualitative phase. However, to ensure equal representation of all item difficulty levels, and to decrease the number of items, the team opted to instead select two items from each error band (2, 3, 8, 9) with a content validity index of one. Given the variety of driving challenges on the spectrum of difficulty, items from different difficulty levels must be adequately represented. Where an error band had more than two items—with an item content validity index of one—only two items were selected by the team. The selection of these items by the team was informed by the theoretical postulates of the conceptual models used for the initial development of the FTDS, i.e., the Precede-Proceed Model of Health Promotion (35), Haddon's matrix (36) and Michon's Model of Driving Behavior (37). The final FTDS-SF constituted 21 items, with those shaded in gray in Table 2. The scale content validity index for the final 21 items was 1.00.

#### Concurrent Validity

**Figure 2:**
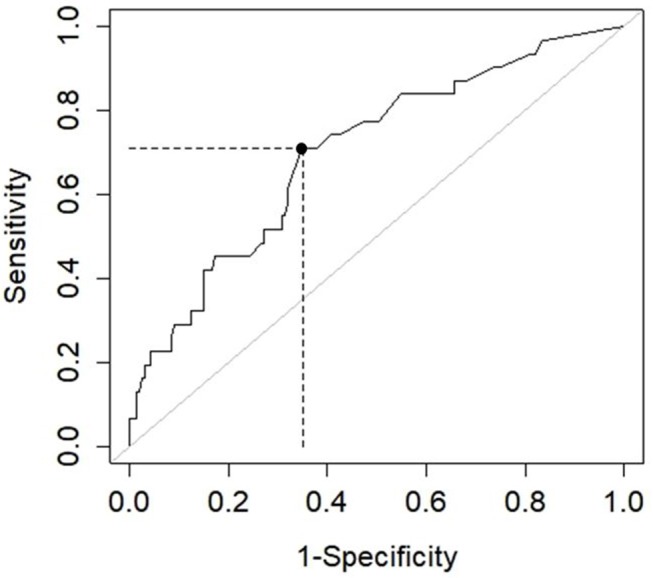
ROC curve for the fitness-to-drive screening measure short-form. AUC = 0.72, *p* < 0.05, CI 95% [0.62–0.82], sensitivity = 0.71, specificity = 0.65, positive predictive value = 0.27, and negative predictive value = 0.92.

For the ROC curve, we used the recalibrated persons measures based on the 21-item FTDS-SF. Older driver logit scores for the 21-item FTDS-SF ranged from 3.52 to 6.73 with an average *SE* of 0.76 logits. The ROC curve depicting the performance of the FTDS-SF to predict pass or fail outcomes of the on-road assessment is presented in Figure 2. The AUC = 0.72 (*p* < 0.05), CI 95% [0.62–0.82], indicating acceptable accuracy (38). The highest *J* index value was 0.36 at the associated cut-point of 3.00 logits. At this cut-point the sensitivity was 0.71 and specificity was 0.65, resulting in 68 (of 200) misclassifications. The PPV = 0.27 and the NPV = 0.92. Using the 3.00 logit cut-point, the thresholds for separating *at-risk drivers* from *routine drivers* was ≤ 1.52 logits, and to separate *routine drivers* from *accomplished drivers*, the logit score was ≤ 4.48.

## Discussion

The purpose of this study was to construct a Rasch based FTDS-SF with clinician input, and to examine it's concurrent validity to actual on-road outcomes.

To select the final items and construct an FTDS-SF with a range of item difficulties (easy to difficult) and driver abilities (low to high), we selected items from each error band. Items within ± 2 *SE* provided the most information for separating drivers and as such were selected for the FTDS-SF. However, not all items with a content validity index of 1 were included, because this would have resulted in a much larger proportion of “easy” items. By combining the content validity approach with team expertise and knowledge from the theoretical models informing the development of the FTDS, the team was able to select a range of items (of varying levels of difficulty) that best fit the goals of the FTDS-SF while still maintaining acceptable scale content validity. This combined approach allowed the team to verify the clinical appropriateness and relevancy of the selected items.

Overall, the 21 item FTDS-SF demonstrated acceptable item and person reliability. Item reliability was 96%, indicating that items of various difficulty levels were precisely located in the item hierarchy. A high item reliability verifies that the FTDS-SF has an item difficulty hierarchy ranging from easy to difficult that can be used to capture a range of driving abilities. Person reliability was 81% signifying that only 19% of the person measure variability was due to measurement error. This indicates that person measures obtained from the 21-item FTDS-SF, have reliable estimates.

The AUC for the FTDS-SF indicated the tool could predict on-road outcomes with acceptable accuracy. At the 3.00 logit cut-point, the sensitivity was higher than specificity. Higher sensitivity suggests more at-risk older drivers are correctly identified. By correctly identifying at-risk drivers, they may become aware of their declining fitness to drive abilities and receive the resources needed to keep them on the road, safer and for longer periods, or, if driving is no longer viable to start conversations about driving cessation.

From the 68 misclassifications, the majority of the older drivers were incorrectly classified as failing (*n* = 59) when they had passed the on-road assessment. Older drivers who become aware of their declining fitness to drive abilities often adjust their driving patterns and behaviors (39). Thus, older drivers misclassified by the FTDS-SF may implement unnecessary self-regulation strategies (e.g., decision not to take a trip) that may affect quality of life.

The NPV of the FTDS-SF was higher than the PPV. Therefore, if the FTDS-SF classifies a driver as an accomplished driver, there is a 92% probability that the driver will also pass the on-road assessment. However, if the FTDS-SF classifies a driver as an at-risk driver, there is only a 27% probability the driver will actually fail the on-road assessment. This suggests that fail classifications must be interpreted with caution, as some of the classifications may be a false (vs. true) positive. The FTDS-SF is therefore best used as a baseline screening measure to identify risk propensity of the driver (i.e., *at-risk driver, routine driver, accomplished driver*) and to initiate conversations about driver fitness. The FTDS may help to more accurately and efficiently identify clients that may require further evaluation or on-road assessments. Specifically, using the 3.00 logit cut-point, older drivers can be separated as at-risk drivers (logit score < 1.52), routine drivers (logit score between 1.52 and 4.48), and accomplished drivers (logit score >4.48).

### Limitations and Strengths

Sample size recommendations for Rasch analysis using *de Ayala's 2:1 rule* suggested this study required a sample of at least 246 participants. However, de Ayala's rule is best suited for evenly distributed responses (40). Because our sample was not evenly distributed, the team used Linacre's *10 responses per category rule* for sample size (40). For content validity analysis the literature suggests using six content experts (29); however, Lynn (29) posits that as few as three content experts (as was the case in this study) can be used to determine content validity.

Compared to classical test theory, Rasch analysis provides many advantages, including that it is sample and test-free. This means a respondent's estimated ability is independent of the sample and measure used (41). Rasch also converts raw non-linear scores to linear scores and thereby enables the researcher to make interval comparison. The method is also robust to missing data (41). Barring the issues with lower sensitivity (than specificity) and number of false positives (59/200), the FTDS-SF may be used as an efficient and valid screening to identify at-risk older drivers and be used as a base for starting conversations about driving.

## Conclusion

The 21-item FTDS-SF demonstrated acceptable validity for identifying at-risk older drivers. Although the FTDS-SF may help to make efficient fitness to drive decisions, the misclassification of 68 drivers remains a concern.

The authors declare that they do not have any affiliations or financial interest in the subject matter or materials discussed in this manuscript.

The University of Florida's Institute for Mobility, Activity, and Participation provided infrastructure and support.

## Author Contributions

SC and SM assisted with data analysis and carried out data interpretation and preparation of manuscript. SR and ML carried out the data analysis, assisted with data interpretation and editing of the manuscript.

### Conflict of Interest Statement

The authors declare that the research was conducted in the absence of any commercial or financial relationships that could be construed as a potential conflict of interest.

## References

[1] Murdock SH Cline ME Zey M Perez D Jeanty PW Population Change in the United States: Socioeconomic Challenges and Opportunities in the Twenty-First Century. New York, NY: Springer (2015). 10.1007/978-94-017-7288-4

[2] Robertson R Vanlaar W. Elderly drivers: Future challenges? Accident Analysis Prevent. (2008) 40:1982–6. 10.1016/j.aap.2008.08.012 19068304

[3] National Highway Traffic Safety Administration Alcohol-Impaired Driving, 2015 Data. Washington, DC: National Highway Traffic Safety Administration (2016). Available online at: https://crashstats.nhtsa.dot.gov/Api/Public/ViewPublication/812350.

[4] National Highway Traffic Safety Administration Traffic Safety Facts 2015: A Compilation of Motor Vehicle Crash Data From the Fatality Analysis Reporting System and the General Estimates System. Washington, DC (2017). Available online at: https://crashstats.nhtsa.dot.gov/Api/Public/Publication/812384

[5] Naumann RB Dellinger AM Kresnow MJ. Driving self-restriction in high-risk conditions: how do older drivers compare to others? J Safety Res. (2011) 42:67–71. 10.1016/j.jsr.2010.12.001 21392632

[6] Quinlan KP Brewer RD Siegel P Sleet DA Mokdad AH Shults RA Alcohol-impaired driving among U.S. adults, 1993-2002. Am J Prevent Med. (2005) 28:346–50. 10.1016/j.amepre.2005.01.006k 15831339

[7] Owsley C Driver Capabilities Paper Presented at the Transportation in an Aging Society: A Decade of Experience Bethesda, MD: Transportation research board (1999).

[8] Centers for Disease Control and Prevention. Older Adult Drivers (2017). Available online at: https://www.cdc.gov/motorvehiclesafety/older_adult_drivers/index.html

[9] Transportation Research Board A taxonomy and terms for stakeholders in senior mobility. Transport Res Circ. (2016) E-C211:1–32. 10.17226/23666

[10] Insurance Institute for Highway Safety - Highway Loss Data Institute Older Drivers: License Renewal Procedures (2018). Available online at: http://www.iihs.org/iihs/topics/laws/olderdrivers?topicName=older-drivers

[11] American Geriatrics Society and Pomidor A Clinician's Guide to Assessing and Counseling Older Drivers, 3rd edn. Washington, DC: National Highway Traffic Safety Administration (2015).

[12] Adams A. Should family physicians assess fitness to drive? Canad Family Phys. (2010) 56:1264–8. 21375059 PMC3001909

[13] Betz ME Dickerson A Coolman T Schold Davis E Jones J Schwartz R. Driving rehabilitation programs for older drivers in the United States. Occup Ther Health Care (2014) 28:306–17. 10.3109/07380577.2014.908336 24971897 PMC4347878

[14] Carr DB Schwartzberg JG Manning L Sempek J. Physician's Guide to Assessing Counseling Older Drivers. Washington, DC (2010). Available online at: http://www.ama-assn.org/ama/pub/physician-resources/public-health/promoting-healthy-lifestyles/geriatric-health/older-driver-safety/assessing-counseling-older-drivers.shtml

[15] Jang RW Man-Son-Hing M Molnar FJ Hogan DB Marshall SC Auger J. Family physicians' attitudes and practices regarding assessments of medical fitness to drive in older persons. J General Intern Med. (2007) 22:531–43. 10.1007/s11606-006-0043-x 17372806 PMC1829420

[16] Marshall S Demmings EM Woolnough A Salim D Man-Son-Hing M. Determining fitness to drive in older persons: a survey of medical and surgical specialists. Canad Geriatr J. (2012) 15:101–19. 10.5770/cgj.15.30 23259024 PMC3516354

[17] Classen S Velozo CA Winter SM Bédard M Wang Y. Psychometrics of the fitness-to-drive screening measure. OTJR (2015) 35:42–52. 10.1177/1539449214561761 26623476

[18] Classen S Wang Y Winter SM Velozo CA Lanford DN Bedard M. Concurrent criterion validity of the Safe Driving Behavior Measure: a predictor of on-road driving outcomes. Am J Occup Ther. (2013) 67:108–16. 10.5014/ajot.2013.005116 23245789 PMC3722666

[19] Classen S Wen PS Velozo C Bédard M Winter SM Brumback B. Psychometrics of the self-report Safe Driving Behavior Measure for older adults. Am J Occup Ther. (2012) 66:233–41. 10.5014/ajot.2012.001834 22394533

[20] Classen S Winter SM Velozo CA Bédard M Lanford D Brumback B. Item development and validity testing for a Safe Driving Behavior Measure. Am J Occup Ther. (2010) 64:296–305. 20437917 10.5014/ajot.64.2.296 PMC2921635

[21] Classen S Winter SM Velozo CA Hannold EM Stakeholder recommendations to refine the Fitness-to-Drive Screening Measure. Open J Occup Ther. (2013) 1:1–14. 10.15453/2168-6408.1054

[22] Classen S Yarney A Monahan M Platek K Lutz A (2015). Rater reliability to assess driving errors in a driving simulator. Adv Transport Stud Sect. B 36:99–108. 10.4399/97888548856608

[23] Winter SM Classen S Bédard M Lutz B Velozo CA Lanford DN Focus group findings for the self-report Safe Driving Behavior Measure. Canad J Occup Ther (2011) 78:72–9. 10.2182/cjot.2011.78.2.2 21560911

[24] Classen S Medhizadah S Fitness-to-Drive Screening Measure: a valid and reliable tool for occupational therapy practice. OT Pract. (2017) 2:19–21.

[25] Classen S Medhizadah S Alvarez L The Fitness-to-Drive Screening Measure: patterns and trends for Canadian users. Open J Occup Ther. (2016) 4:1–17. 10.15453/2168-6408.1227

[26] Medhizadah S Classen S Johnson AM. Constructing the 32-item fitness-to-drive screening measure. OTJR (2018) 38:89–95. 10.1177/1539449217741136 29126376

[27] Fisher WP Robustness and invariance. Rasch Measure Trans. (1993) 7:295.

[28] Fisher WP The rasch alternative. Rasch Measure Trans. (1996) 9:466–7.

[29] Lynn MR. Determination and quantification of content validity. Nurs Res. (1986) 35:382–5. 10.1097/00006199-198611000-00017 3640358

[30] del Toro CM Bislick LP Comer M Velozo C Romero S Gonzalez RLJ. Development of a short form of the Boston naming test for individuals with aphasia. J Speech Lang Hear Res. (2011) 54:1089–100. 10.1044/1092-4388(2010/09-0119) 21173387

[31] Linacre JM. Optimizing rating scale category effectiveness. J Appl Measure. (2002) 3:85–106. 11997586

[32] Linacre JM Winsteps® Rasch Measurement Computer Program User's Guide. Beaverton, OR: Winstep (2012).

[33] Streiner DL Cairney J. What's under the ROC? An introduction to receiver operating characteristics curves. Canad J Psychiatry (2007) 52:121–8. 10.1177/070674370705200210 17375868

[34] Youden WJ. Index for rating diagnostic tests. Cancer (1950) 3:32–5. 10.1002/1097-0142(1950) 15405679

[35] Green LW Toward cost–benefit evaluations of health education: some concepts, methods, and examples. Health Educ Monogr. (1974) 2:34–64. 10.1177/10901981740020S106

[36] Haddon WJ. A logical framework for categorizing highway safety phenomena and activity. J Trauma (1972) 12:193–207. 10.1097/00005373-197203000-00002 5012817

[37] Michon JA A critical view of driver behavior models: What do we know, what should we do? In: Evans EL Schwing R editors. Human Behavior and Traffic Safety New York, NY: Plenum (1985). p. 485–520.

[38] Hosmer DW Lemeshow S Applied Logistic Regression 2nd edn. New York, NY: John Wiley and Sons (2000).

[39] Molnar LJ Eby DW Zhang L Zanier N St Louis RM, Kostyniuk LP. Self-Regulation of Driving by Older Adults: A Synthesis of the Literature and Framework for Future Research. AAA Foundation for Traffic Safety (2015). p. 1–48.

[40] Willse JT Polytomous rasch models in counselling assessment. Measure Eval Counsel Dev. (2017) 50:248–55. 10.1080/07481756.2017.1362656

[41] Velozo CA Seel RT Magasi S Heinemann AW Romero S. Improving measurement methods in rehabilitation: core concepts and recommendations for scale development. Arch Phys Med Rehab. (2012) 93:S154–63. 10.1016/j.apmr.2012.06.001 22840881

