# Corrigendum: Construction and Validation of the 21 Item Fitness-to-Drive Screening Measure Short-Form

**DOI:** 10.3389/fpubh.2019.00086

**Published:** 2019-04-16

**Authors:** Sherrilene Classen, Shabnam Medhizadah, Sergio Romero, Mi Jung Lee

**Affiliations:** ^1^Department of Occupational Therapy, University of Florida, Gainesville, FL, United States; ^2^Center of Innovation on Disability & Rehabilitation Research, United States Department of Veterans Affairs, Gainesville, FL, United States

**Keywords:** aging, proxy raters, decision support system, automobile driving, fitness to drive

In the original article, there was an error. The final FTDS-SF that constituted the 21 items was referred to as “bolded” in Table 2, however it should have stated that the 21 items are “shaded in gray” in Table 2 instead.

A correction has been made to the **Results**, subsection **Qualitative Analysis**:

“The remaining error bands (2, 3, 8, 9), had atleast one item that had a content validity index of one. As shown in Table 2, initially 11 items (i.e., item #13, 21, 29, 31, 32, 37, 38, 48, 49, 51, 52) with an item content validity index of 1 were selected through the content validity approach. This resulted in an item bank of 24 items, with 13 items selected from the quantitative phase and 11 items from the qualitative phase. However, to ensure equal representation of all item difficulty levels, and to decrease the number of items, the team opted to instead select two items from each error band (2, 3, 8, 9) with a content validity index of one. Given the variety of driving challenges on the spectrum of difficulty, items from different difficulty levels must be adequately represented. Where an error band had more than two items—with an item content validity index of one—only two items were selected by the team. The selection of these items by the team was informed by the theoretical postulates of the conceptual models used for the initial development of the FTDS, i.e., the Precede-Proceed Model of Health Promotion (35), Haddon's matrix (36) and Michon's Model of Driving Behavior (37). The final FTDS-SF constituted 21 items, with those shaded in gray in Table 2. The scale content validity index for the final 21 items was 1.00.”

The authors apologize for this error and state that this does not change the scientific conclusions of the article in any way. The original article has been updated.

